# Immune- and miRNA-response to recombinant interferon beta-1a: a biomarker evaluation study to guide the development of novel type I interferon- based therapies

**DOI:** 10.1186/s40360-015-0025-x

**Published:** 2015-09-22

**Authors:** Martin Coenen, Annette Viktoria Hinze, Martin Mengel, Christine Fuhrmann, Bastian Lüdenbach, Julian Zimmermann, Verena Dykstra, Rolf Fimmers, Roberto Viviani, Julia Stingl, Stefan Holdenrieder, Marcus Müller, Gunther Hartmann, Christoph Coch

**Affiliations:** Clinical Study Core Unit, Study Center Bonn (SZB), University of Bonn, Sigmund-Freud-Str. 25, 53127 Bonn, Germany; Institute of Clinical Chemistry and Clinical Pharmacology, University of Bonn, Sigmund-Freud-Str. 25, 53127 Bonn, Germany; Federal Institute for Drugs and Medical Devices, Kurt-Georg-Kiesinger-Allee 3, 53175 Bonn, Germany; Institute of Medical Biometrics, Informatics and Epidemiology, University of Bonn, Sigmund-Freud-Str. 25, 53127 Bonn, Germany; Department of Psychiatry and Psychotherapy III, University of Ulm, Ulm, Germany; Deparment of Neurology, University of Bonn, Sigmund-Freud-Str. 25, 53127 Bonn, Germany

**Keywords:** Interferon-beta, Immune response, Tumor immune therapy, Multiple sclerosis, Dose calculation, Phase I study, RIG-I, miRNA, Cytokines

## Abstract

**Background:**

The innate immune receptor RIG-I detects viral RNA within the cytosol of infected cells. Activation of RIG-I leads to the induction of antiviral cytokines, in particular type I interferon, the inhibition of a T(H)17 response as well as to the suppression of tumor growth. Therefore, RIG-I is a promising drug target for the treatment of cancer as well as multiple sclerosis. A specific ligand for RIG-I is currently in preclinical testing. The first-in-human trial will need to be carefully designed to avoid an overshooting cytokine response. Therefore, the ResI study was set up to analyze the human immune response to standard treatment with recombinant interferon-beta to establish biomarkers for safety and efficacy of the upcoming first-in-human trial investigating the RIG-I ligand.

**Methods/Design:**

ResI is a single center, prospective, open label, non-randomized phase I clinical trial. Three different cohorts (20 healthy volunteers, 20 patients with RRMS and ongoing interferon-beta treatment and 10 patients starting on interferon-beta) will receive standard interferon-beta-1a therapy for nine days. The study will be conducted according to the principles of the german medicinal products act, ICH-GCP, and the Declaration of Helsinki on the phase I unit of the Institute of Clinical Chemistry and Clinical Pharmacology and in the Department of Neurology, both University Hospital Bonn. Interferon-beta-induced cytokine levels, surface marker on immune cells, mRNA- and miRNA-expression as well as psychometric response will be investigated as target variables.

**Discussion:**

The ResI study will assess biomarkers in response to interferon-β treatment to guide the dose steps within the first-in-human trial with a newly developed RIG-I ligand. Thus, ResI is a biomarker study to enhance the safety of the clinical development of a first-in-class compound. The data can additionally be used for the development of other therapies based on type I interferon induction such as TLR ligands. Moreover, it will help to understand the interferon-beta induced immune response in a controlled *in vivo* setting in the human system.

**Trial registration:**

clinicaltrials.gov ID NCT02364986

## Background

Retinoic acid inducible gene-I (RIG-I) is a pattern recognition receptor (PRR) of the innate immune system, broadly expressed in immune - but also in non-immune cells, including tumor cells [1–3]. RIG-I is a sensor for pathogenic RNA, detecting 5’-triphosphorylated RNA (3pRNA) in the cytosol of infected cells [4, 5], crucially important to protect from different RNA viruses [2, 6]. Activation of RIG-I leads to a broad antiviral immune response, including strong induction of type I interferon (IFN), to eradicate the invading pathogen. A potent benefit of therapeutic activation of RIG-I was described recently in different indications: i) 3pRNA suppresses tumor growth by triggering apoptosis specifically in tumor but not in non-malignant cells as well as by inducing an anti-tumor immune response, including type I IFN induction and NK cell activation [7–10]. ii) Activation of RIG-I inhibits the viral replication and is therefore protective in viral diseases such as Hepatitis B [11] and influenza infection (unpublished data). iii) Activation of RIG-I in mice with induced experimental autoimmune encephalomyelitis, the animal model for MS, significantly ameliorates autoimmune inflammation, decreases demyelination and improves the clinical outcome by repressing the maintenance and expansion of T(H)1 and T(H)17 cells [12]. As in all these indications an unmet therapeutic need exists, triggering of RIG-I by a synthetic short 3pRNA-ligand is a promising new therapeutic approach with broad clinical implication [1]. A specific RNA ligand for RIG-I is currently under preclinical evaluation. Even though we identified relevant animal species for preclinical toxicity testing (unpublished data) the RIG-I ligand bears characteristics making increased caution in the first-in-human trial necessary as outlined in the European Medicines Agency (EMA) “Guideline on strategies to identify and mitigate risks for first-in-human clinical trials with investigational medicinal products” (EMEA/CHMP/SWP/28367/07): i) as first-in-class compound it is a novel mechanism of action, involving multiple signalling pathways [13] ii) it is an activating mechanism and iii) the RIG-I ligand acts on the immune system inducing the release of antiviral and pro-inflammatory cytokines [1, 4, 5]. To minimize the probability of a possible overshooting cytokine induction in the first-in-man phase I trial, thorough preclinical testing needs to be performed. In addition, biomarkers indicating activity or toxicity of RIG-I agonisation would be of great value to safely guide the first-in-man trial. Moreover, such biomarkers could be used as surrogate parameter to gather first data on the potential efficacy of a RIG-I ligand, starting in the first-in-man trial and later on in more advanced stages of the clinical development. Type I IFN serum level as well as the dependent immune response will bear suitable information for dose guiding of the RIG-I ligand since type I IFN is a key cytokine induced by RIG-I [4, 14]. To give an orientation on a type I IFN level that is potentially therapeutic active, the effect of standard interferon treatment as routinely applied in multiple sclerosis can be used. As soon as the type I IFN response induced by a certain dose of the RIG-I ligand reaches the levels induced by standard treatment with recombinant IFN-β, this level can be regarded as a potentially effective dose. With this information the dose steps within the phase I study can be carefully guided, side effects better monitored and there will be no need to explore the maximal tolerable dose.

For this purpose, the ResI study was set up as a clinical trial aiming to establish a biomarker for the safety and efficacy of the RIG-I ligand, a novel therapeutic entity currently in preclinical testings. Since studies of the RIG-I ligand will start in healthy volunteers and will be continued in MS patients both populations are included in this study as they could show significant differences in response to IFN-β. Additionally, this comprehensive, prospective analysis of the IFN-β dependent immune activation and changes in miRNA expression in this controlled setting will help to understand the physiological response to IFN-β in healthy volunteers and in MS patients.

## Methods/Design

ResI is a single center, prospective, open label, non-randomized phase I clinical trial. Three different cohorts (healthy volunteers, patients with RRMS and ongoing IFN-β treatment and patients with RRMS starting on IFN-β treatment) will receive three applications of standard IFN-β therapy for nine days. As a comparison between pre- and post-treatment is performed, no placebo group is planned. The study will be conducted according to the principles of the german medicinal products act, ICH-GCP, and the Declaration of Helsinki on the phase I unit of the Institute of Clinical Chemistry and Clinical Pharmacology and in the Department of Neurology, both University Hospital Bonn. The local ethics committee and the German competent authorities (Federal Institute for Drugs and Medical Devices) already approved this study. The Clinical Study Core Unit will perform the clinical procedures, monitoring, safety management, data management, and data analysis of the study. The Institute of Clinical Chemistry and Clinical Pharmcology, that includes the central laboratory of the University Hospital Bonn will perform the routine as well as experimental laboratory analysis.

### Primary objective

The primary objective of this phase I trial is to determine IFN-β protein level as well as IFN-β-dependent mRNA and protein expression in healthy volunteers and in RRMS patients continuing or starting on IFN-β treatment (including CXCL10, MxA, RIG-I).

### Secondary objectives

Secondary objectives of this trial are the analysis of the miRNA- and gene-expression pattern in response to recombinant IFN-β in peripheral blood mononuclear cells and serum of healthy volunteers as well as RRMS patients, the comparison of the response to IFN-β between healthy volunteers, patients with RRMS naïve to IFN-β treatment and RRMS-patients with established IFN-β treatment, correlation of side effects of IFN-β treatment with immune- or miRNA-response to IFN-β. Potential central nervous adverse drug effects such as emotional changes associated with IFN-β treatment (depression, anxiety, impulsivity) will be assessed by psychometric testings and noninvasive neuroimaging techniques.

### Inclusion criteria

Three different cohorts will be studied: 1.) Healthy volunteers naïve for IFN-β treatment. 2.) patients with relapsing-remitting MS (RRMS) according to McDonald's criteria and baseline expanded disability status scale (EDSS) score from 0 to 6.0 starting on IFN-β treatment according to routine clinical criteria. 3.) Patients as described above but already on IFN-β treatment. Additional inclusion criteria applicable for all individuals in this study: age between 18 and 65 years, adequate bone marrow-, renal-, hepatic- and clotting-function. Male and female patients with reproductive potential have to use an approved contraceptive method and a negative serum pregnancy test must be obtained prior to treatment start in female patients with childbearing potential. All individuals need to have signed the informed consent form.

### Exclusion criteria

The main exclusion criteria comprise of: known allergy or hypersensitivity to IFN-β or ingredients of the injection solution; condition or disease which at the investigator’s discretion do not fit with the study; known or persistent abuse of medication, drugs or alcohol; current use of cortisone preparation; subjects with reproductive potential who do not accept to use contraception during the trial and 3 months thereafter or women who are pregnant or breast-feeding; prior malignancy except for adequately treated carcinoma in situ of the cervix or non-melanoma skin cancer unless prior malignancy was diagnosed and definitively treated at least 5 years previously with no subsequent evidence of recurrence the subject at the discretion of the investigator; prior chemotherapy, systemic or local treatment with DNA-damaging and immune-modulating agents, tyrosine kinase inhibitors or anti-angiogenic agents for any cancer; history of major depression, suicide attempt in the past, ongoing suicidal thoughts; history of epileptic seizures under medical therapy with antiepileptic drugs; cardiac insufficiency (NYHA III or IV) cardiomyopathy, significant cardiac dysrhythmia, unstable or advanced ischemic heart disease, or significant hypertension at rest (blood pressure > 180/110 mmHg); HIV, Hepatitis B or C infection or any relevant infectious disease which might interfere with the study procedures and results (at the discretion of the investigator).

### Therapy

Three different cohorts will be treated in this study: healthy volunteers, patients with RRMS and ongoing IFN-β treatment and patients with RRMS starting on IFN-β treatment. Patients and healthy volunteers will receive three doses of 44 μg open-label Interferon-β-1a (Rebif®) within nine days as recommended in the standard treatment schedule. MS patients already on IFN-β treatment will only receive one dose of IFN-β while included in this study. Dose modification or adjustments for either group are not intended by the trial protocol. After the study is finished MS patients will be treated at the discretion of the responsible physician.

At or before the screening visit, each patient has to provide written informed consent to the study. The screening consists of reviewing inclusion and exclusion criteria, demographic variables, medical history, concomitant medication, physical and neurological examination and vital signs, electrocardiogram (ECG) and laboratory tests are obtained by a study nurse and the treating physician. MRI, EDSS scoring and psychometric tests are performed. Laboratory tests include complete blood count, serum chemistry, coagulation, drug screening, pregnancy test and urine analysis.

At baseline visit vital signs and ECG as well as the occurrence of adverse events and changes of the concomitant medication are documented. Screening and baseline visit are performed within 14 days. Then IFN-β-1a treatment is started and blood for the analysis of cytokine serum levels, miRNA- and mRNA-expression as well as experimental immune evaluation is collected at five different times within 24 h. Thereupon, the study is finished for MS patients that have been already on IFN-β treatment. Healthy volunteers as well as MS patients that have been started on IFN-β will continue with study visits at days 3, 5 and 8 with documentation of adverse events and concomitant medication and collection of further blood samples for analysis of immune activation. At day nine healthy volunteers as well as MS patients will have the final visit with the same assessments as described for the screening and baseline visit, including magnetic resonance imaging (MRI) assessment. Additional, blood for analysis of immune activation is collected.

### Magnetic resonance imaging

To characterize neurobiological correlates of the action of IFN-β, changes in base perfusion levels, in limbic reactivity to emotionally laden stimuli, in the neural response to potential rewards, and their association with psychometric parameters will be assessed in healthy volunteers through noninvasive neuroimaging techniques. All MRI scans are performed according to a standardized MRI protocol and will be assessed centrally. MRI scans of the brain will be performed only in the healthy volunteers at the screening and at the final visit. Functional neuroimaging of limbic reactivity will be performed to analyze both emotional reactivity and the capacity of participants to down-regulate it.

### Statistical analysis

The **sample size** is set on 20 healthy volunteers and 20 patients with established IFN-β treatment and 10 MS patients naïve to IFN-β treatment. Since this is a study to gain information about the distribution of the expression values in these patients, this sample size is not based on statistical considerations.

**Primary target variables** of the study is the estimation of changes in serum level of type I IFN and type I IFN-dependent proteins (e.g. CXCL10) as well as of changes in mRNA expression of type I IFN-dependent genes (e.g. CXCL10, MxA and RIG-I) after a dose of IFN-β treatment in healthy volunteers and in patients with RRMS. For this purpose, the median of the levels will be calculated separately for the two study groups. 95 % confidence intervals for the medians will be calculated using bootstrap. As additional descriptive parameters mean, standard deviation, minimum, maximum and the quartiles of the distribution will be calculated for each group and variable. A comparison of the expression levels between patients and controls will be performed with a two-sided Mann–Whitney-Wilcoxon test. These tests are regarded as descriptive analysis, their result are reported as p-values.

## Discussion

Mimicking a viral infection by activating pattern recognition receptors (PRR) of the innate immune system is an interesting therapeutic approach, tested for a variety of indications [1, 15, 16]. Apart from the toll-like receptor family, the retinoic acid inducible gene-I (RIG-I) is a particular promising candidate for this strategy. RIG-I is suggested as drug target in a broad range of indications including viral infections, cancer and autoimmune disease such as multiple sclerosis [1]. RIG-I is ubiquitously expressed in non-malignant but also malignant cells and leads not only to the release of antiviral cytokines (e.g. type I IFN) and NK cell activation but also to apoptosis induction in cancer but not in non-malignant cells [7–11]. Moreover, RIG-I activation can suppress a T(H)17 response *in vivo* [12]. Thus, the immediate and comprehensive immune response orchestrated by RIG-I seems to be of great potential in a variety of diseases with a still unmet medical need. But it needs to be taken into account that all these mechanisms of actions are not only beneficial but can also cause side effects depending on the dose. Therefore, the immune activation by RIG-I needs to be sufficiently understood and well balanced. The disastrous outcome of the TeGenero trial unintentionally demonstrated the consequences of an underestimated activating immune effect leading to a cytokine storm [17]. As a consequence the EMA released a guideline describing risk factors to be considered before starting a first-in-human phase I trial (EMEA/CHMP/SWP/28367/07). As the RIG-I ligand represents a new mode of action, is activating in nature and turns on different immune pathways, this therapeutic approach has multiple aspects of high risk compounds. Therefore, the preclinical data need to be gathere carefully and scientifically driven to obtain a comprehensive understanding of the potential outcomes of the RIG-I ligand administration. To further reduce the risk of the upcoming phase I studies with the new RIG-I ligand, we decided to design a clinical study to add human *in vivo* data that are suitable to function as a biomarker for the immune activation induced by RIG-I. To this end, we will comprehensively measure the immune response induced by standard treatment with recombinant IFN-β, including protein and mRNA expression of known IFN-β dependent genes (e.g. CXCL10, MxA, RIG-I), miRNA expression pattern, genome wide mRNA expression. In the subsequent phase I studies with the RIG-I ligand these information will be used to judge whether the induced type I IFN is below or above standard treatment with recombinant IFN-β. In addition, as emotional changes are described for type I IFN [18–21], we will perform functional MRI scans to screen for early effects in order to learn how this side effect can be detected early upon exposure to a RIG-I ligand. Therefore, the study presented here will provide crucial data to safely guide the application of the RIG-I ligand. To rule out differences in the response to IFN-β we will include both healthy volunteers as the first population within the clinical testing being exposed to the RIG-I ligand as well as patients with relapsing-remitting multiple sclerosis as patients meant to be treated with the RIG-I ligand later in development. Although numerous effects of type I IFN on the immune system are well established since decades, the results from the literature cannot be used here for different reasons: data obtained in animal or *in vitro* systems do not necessarily reflect the *in vivo* situation in humans, the complete set of read outs were not investigated in a single comprehensive study, it is unclear how repeatable the assays are that were used, and studies were often done in patients but not healthy volunteers with confounding factors such as concomitant medications. In addition, potential adverse neurological drug effects have to be assessed *in vivo* in humans without confounding factors of neurological disease symptoms. Therefore, a lot of emphasis is laid on the quantitative measurement of potential emotional or cognitive changes due to the exposure to IFN-β in healthy humans using MRI techniques. To be technically able to transfer the results in the biomarker study to the phase I studies with the RIG-I ligand, the main read outs used here (ELISA and qPCR) will be validated on repeatability, precision, pre-assay effects, dilution linearity and adequate controls and standards will be generated and included that can be used for comparison in future studies. Nevertheless, information obtained here will bear certain limitations: activation of RIG-I leads to a profound induction of type I IFN but also induces additional pathways and cytokines such as pro-inflammatory cytokines (e.g. IL-6) and IFN-γ (unpublished data) not mimicked by application of IFN-β alone. Additionally, the time course of type I IFN induction caused by activation of RIG-I is not necessarily mirrored by application of the recombinant protein. Therefore, the results of this biomarker study need to be interpreted in connection with the complete preclinical program and are not meant to substitute for it. But taking these limitations into account, this study is a rare, maybe unique approach to define biomarkers in the human system *in vivo* that increases the safety of the clinical development of a drug before the actual compound is given the first time to healthy subjects. The data obtained here can be additionally used to guide other treatments also involving induction of type I IFN, such as ligands for TLR7 or 9 (as intended effect or unwanted side effect, e.g. siRNA) [15, 16, 22, 23]. Therefore, this study proposes a way, how the safety of a first-in-man phase I clinical trial can be enhanced in addition to the principles outlined in the current guidelines of drug development. As additional benefit, this study is suitable to find new factors being induced by type I IFN in the human system *in vivo*, namely in- or decreased miRNA, since studies investigating the effects of type I IFN in a very controlled human *in vivo* system in healthy volunteers are rare.

## Abbreviations

MS: multiple sclerosis
RRMS: rapid-relapsing multiple sclerosis
IFN: Interferon
IFN-β: Interferon-beta
EDSS: Expanded disability status scale
ECG: Electrocardiogram
MRI: Magnetic resonance imaging
RIG-I: Retinoic acid inducible gene-I
PRR: Pattern recognition receptor
3pRNA: 5’-triphosphorylated RNA
EMA: European Medicines Agency

## References

[1] Barchet W, Wimmenauer V, Schlee M, Hartmann G (2008). Accessing the therapeutic potential of immunostimulatory nucleic acids. Curr. Opin. Immunol..

[2] Kawai T, Akira S (2006). Innate immune recognition of viral infection. Nat. Immunol..

[3] Kawai T, Akira S (2009). The roles of TLRs, RLRs and NLRs in pathogen recognition. Int. Immunol..

[4] Hornung V, Ellegast J, Kim S, Brzózka K, Jung A, Kato H, Poeck H, Akira S, Conzelmann K-K, Schlee M, Endres S, Hartmann G (2006). 5'-Triphosphate RNA is the ligand for RIG-I. Science.

[5] Schlee M, Roth A, Hornung V, Hagmann CA, Wimmenauer V, Barchet W, Coch C, Janke M, Mihailovic A, Wardle G, Juranek S, Kato H, Kawai T, Poeck H, Fitzgerald KA, Takeuchi O, Akira S, Tuschl T, Latz E, Ludwig J, Hartmann G (2009). Recognition of 5' triphosphate by RIG-I helicase requires short blunt double-stranded RNA as contained in panhandle of negative-strand virus. Immunity.

[6] Kato H, Takeuchi O, Sato S, Yoneyama M, Yamamoto M, Matsui K, Uematsu S, Jung A, Kawai T, Ishii KJ, Yamaguchi O, Otsu K, Tsujimura T, Koh C-S (2006). Differential roles of MDA5 and RIG-I helicases in the recognition of RNA viruses. Nature.

[7] Poeck H, Besch R, Maihoefer C, Renn M, Tormo D, Morskaya SS, Kirschnek S, Gaffal E, Landsberg J, Hellmuth J, Schmidt A, Anz D, Bscheider M, Schwerd T, Berking C, Bourquin C, Kalinke U, Kremmer E, Kato H, Akira S, Meyers R, Häcker G, Neuenhahn M, Busch D, Ruland J, Rothenfusser S, Prinz M, Hornung V, Endres S, Tüting T, Hartmann G (2008). 5'-Triphosphate-siRNA: turning gene silencing and Rig-I activation against melanoma. Nat. Med..

[8] Besch R, Poeck H, Hohenauer T, Senft D, Häcker G, Berking C, Hornung V, Endres S, Ruzicka T, Rothenfusser S, Hartmann G (2009). Proapoptotic signaling induced by RIG-I and MDA-5 results in type I interferon-independent apoptosis in human melanoma cells. J. Clin. Invest..

[9] Glas M, Coch C, Trageser D, Daßler J, Simon M, Koch P, Mertens J, Quandel T, Gorris R, Reinartz R, Wieland A, Von Lehe M, Pusch A, Roy K, Schlee M, Neumann H, Fimmers R, Herrlinger U, Brüstle O, Hartmann G, Besch R, Scheffler B (2013). Targeting the cytosolic innate immune receptors RIG-I and MDA5 effectively counteracts cancer cell heterogeneity in glioblastoma. Stem Cells.

[10] Kübler K, Pesch CT, Gehrke N, Riemann S, Daßler J, Coch C, Landsberg J, Wimmenauer V, Pölcher M, Rudlowski C, Tüting T, Kuhn W, Hartmann G, Barchet W (2011). Immunogenic cell death of human ovarian cancer cells induced by cytosolic poly(I:C) leads to myeloid cell maturation and activates NK cells. Eur J Immunol.

[11] Ebert G, Poeck H, Lucifora J, Baschuk N, Esser K, Esposito I, Hartmann G, Protzer U (2011). 5' Triphosphorylated small interfering RNAs control replication of hepatitis B virus and induce an interferon response in human liver cells and mice. Gastroenterology.

[12] Dann A, Poeck H, Croxford AL, Gaupp S, Kierdorf K, Knust M, Pfeifer D, Maihoefer C, Endres S, Kalinke U, Meuth SG, Wiendl H, Knobeloch K-P, Akira S, Waisman A, Hartmann G, Prinz M (2012). Cytosolic RIG-I-like helicases act as negative regulators of sterile inflammation in the CNS. Nat. Neurosci..

[13] Kawai T, Akira S (2008). Toll-like receptor and RIG-I-like receptor signaling. Ann. N. Y. Acad. Sci..

[14] Schlee M, Barchet W, Hornung V, Hartmann G (2007). Beyond double-stranded RNA-type I IFN induction by 3pRNA and other viral nucleic acids. Curr. Top. Microbiol. Immunol..

[15] Krieg AM (2006). Therapeutic potential of Toll-like receptor 9 activation. Nat Rev Drug Discov.

[16] Hennessy EJ, Parker AE, O'Neill LAJ (2010). Targeting Toll-like receptors: emerging therapeutics?. Nat Rev Drug Discov.

[17] Suntharalingam G, Perry MR, Ward S, Brett SJ, Castello-Cortes A, Brunner MD, Panoskaltsis N (2006). Cytokine storm in a phase 1 trial of the anti-CD28 monoclonal antibody TGN1412. N. Engl. J. Med..

[18] Plosker GL (2011). Interferon-β-1b: a review of its use in multiple sclerosis. CNS Drugs.

[19] Reyes-Vázquez C, Prieto-Gómez B, Dafny N (2012). Interferon modulates central nervous system function. Brain Res..

[20] Fontana RJ (2000). Neuropsychiatric toxicity of antiviral treatment in chronic hepatitis C. Dig Dis.

[21] Udina M, Castellví P, Moreno-España J, Navinés R, Valdés M, Forns X, Langohr K, Solà R, Vieta E, Martín-Santos R (2012). Interferon-induced depression in chronic hepatitis C: a systematic review and meta-analysis. J Clin Psychiatry.

[22] Hornung V, Barchet W, Schlee M, Hartmann G. RNA recognition via TLR7 and TLR8. In: Bauer S, Hartmann G, editors. Handb Exp Pharmacol Toll-like Receptors (TLRs) and Innate Immunity. Springer Berlin Heidelberg. 2008. p. 71–86. 10.1007/978-3-540-72167-3_4 18071655

[23] Hornung V, Guenthner-Biller M, Bourquin C, Ablasser A, Schlee M, Uematsu S, Noronha A, Manoharan M, Akira S, de Fougerolles A, Endres S, Hartmann G (2005). Sequence-specific potent induction of IFN-alpha by short interfering RNA in plasmacytoid dendritic cells through TLR7. Nat. Med..

